# Identify key transcript factors of adipocyte differentiation in abdominal fat of broilers based on ATAC-seq and RNA-seq

**DOI:** 10.1016/j.psj.2025.105096

**Published:** 2025-03-24

**Authors:** Xiaoying Liu, Chaohui Wang, Xi Sun, Zhihao Qiao, Xiaojun Yang, Yanli Liu

**Affiliations:** College of Animal Science and Technology, Northwest A&F University, Yangling, PR China

**Keywords:** Abdominal fat, Adipocyte differentiation, ATAC-seq, RNA-seq

## Abstract

Intensive breeding has resulted in excessive deposition of abdominal fat tissue (AFT) in broilers, leading to significant economic loss in the poultry industry. Understanding the molecular mechanisms underlying AFT development is essential for informed breeding strategies. In the current study, we elucidated dynamic changes of chromatin accessibility and transcriptional reprogramming in AFT at D14 and D42 in broilers based on integrated analysis of RNA-seq and ATAC-seq. RNA-seq analysis manifested significant transcriptional differences in AFT development, identifying 1323 up- and 1285 down-regulated differential expression genes (DEGs) as well as 63 up- and 58 down-regulated transcription factors (TFs) at D42 compared to those at D14. Kyoto Encyclopedia of Genes and Genomes (KEGG) analysis of total DEGs revealed significant enrichment in pathways related to DNA replication, cell adhesion molecules, cell cycle, p53 signaling pathway, fatty acid degradation, fatty acid biosynthesis and steroid biosynthesis. Gene set enrichment analysis (GSEA) further indicated that autophagy, MAPK signaling pathway and inositol phosphate metabolism were up-regulated at D42 compared to D14, whereas cell cycle, DNA replication and steroid biosynthesis were down-regulated. Additionally, ATAC-seq analysis identified 394 gain and 1195 loss differentially accessible peaks (DPs) in AFT between D14 and D42, associated with 319 and 905 genes, respectively. These gain or loss genes were enriched in p53 signaling pathway, PPAR signaling pathway, fat digestion and absorption, FoxO signaling pathway and glycerol lipid metabolism. Integration analysis of ATAC-seq and RNA-seq data revealed 25 up-regulated and 75 down-regulated DEGs overlapping with genes linked to gain and loss DPs, respectively. Notably, ACACA, *SCD, SREBF1*and *KLF9* exhibited significantly lower expression at D42 compared to D14. DNA motifs analysis identified *NFIX* and *MYB* as loss motifs, overlapping with down-regulated TFs, suggesting their potential role in AFT regulation. Furthermore, *MYB* and *NFIX* exhibited potential binding sites in the promoter regions of lipid metabolism-related genes (*ELOVL6, PPARγ, FABP4, ACACA* and *SCD*). Overall, these results will provide a theoretical basis for investigating the epigenetic modification and transcriptional regulation of AFT development in broilers.

## Introduction

Continuous improvement of genetic breeding and artificial selection has accelerated growth rates and increased body weight in broilers (Chen et al., 2023; Miyumo et al., 2024), while concomitantly leading to excessive abdominal fat deposition (Wang et al., 2017), which adversely affects meat quality and reduces feed conversion ratio (Wu et al., 2023). Traditional breeding methods have faced severe challenges in reducing abdominal fat deposition due to the positive genetic and phenotypic correlation between abdominal fat tissue (AFT) mass and body weight (Tunim et al., 2021; Zerehdaran et al., 2004). Therefore, it is necessary to elucidate the potential mechanisms of AFT development in order to decrease AFT deposition and ultimately breed low-fat broilers.

RNA-seq has been widely used to identify differentially expressed genes associated with lipid synthesis or lipolysis (Xing et al., 2019; Liu et al., 2019), and many studies also greatly enhanced the understanding of fat deposition characteristics (Xing et al., 2020; Yuan and Lu, 2021). But the specific molecular mechanisms remain incompletely understood (Liang et al., 2020). Therefore, new methods are urgently needed to elucidate the gene regulatory network. Chromatin accessibility is both a result of epigenetic modification and a prerequisite for gene transcription. ATAC-seq can reveal chromatin accessibility and identify active regulatory elements, such as promoters and enhancers (Fc et al., 2022). Thus, integrating RNA-seq and ATAC-seq has been used to provide a comprehensive analysis of the molecular mechanisms underlying intramuscular fat content in pigs (Xu et al., 2022), furthermore uncovering the gene regulatory networks of lipid metabolism. In addition, previous studies have utilized a combination of ATAC-seq and RNA-seq to demonstrate that chromatin accessibility in adipocyte was closely correlated with gene expression during adipocytes differentiation of yaks, identifying transcription factors (TFs) involved in adipogenesis, including *FOSL2, JUND, FOS* and *JUNB* (Zhang et al., 2022b). Additionally, chromatin accessibility profiling was mapped during bovine myoblast proliferation and differentiation by ATAC-seq and RNA-seq, and revealed specific enrichment of TFs at various stages of myogenesis, such as *MAFF, ZNF384* and *KLF6* (Li et al., 2022). However, the mechanism on abdominal fat development is unclear in broilers, which is required to further investigate chromatin accessibility profiling and potential TFs.

Fat tissue development results from hyperplasia and hypertrophy. However, the number of adipocytes tended to stabilize at later stage of growth, and fat deposition primarily relied on the hypertrophy of adipocytes (Nematbakhsh et al., 2021). Our previous study investigated the dynamic changes of abdominal fat cell during of broilers and reported D14 might be the crucial physiological stage for adipocyte hypertrophy, while the differentiation capacity of adipocyte gradually declined between D14 and D42 (Liu et al., 2023). Considering that D14 represented the early stage of AFT deposition, while D42 marked the final stage of AFT accumulation, this study elucidated the developmental patterns of AFT in broilers during the late growth stage based on integrated analysis of RNA-seq and ATAC-seq. The aim was to reveal chromatin accessibility changes associated with adipocytes differentiation and to identify key TFs driving AFT deposition. This study will establish a novel theoretical basis for investigating the epigenetic modification and transcriptional regulation of AFT development in broilers, which is valuable for scientific guidance to breeding low-fat broilers.

## Materials and methods

All broilers and experimental protocols in this study were approved for implementation by the Animal Care and Use Committee of the college of Animal Science and Technology of the Northwest A&F University (Shaanxi, China, Permit Number: DK202123).

### Animals and sample collection

At the Experimental Teaching Center of Animal Science in the Northwest A&F University, a total of 100 one-day Arbor Acres broiler chickens sourced from Xi'an DaCheng Poultry Co., Ltd. (Xianyang, China) were given commercial diet and water. At both 14 (D14) and 42 days (D42) of age, 10 broilers were randomly chosen and euthanized by cervical dislocation. Subsequently, dissection was performed to collect abdominal fat, which was frozen using liquid nitrogen and stored at −80°C.

### Library construction and sequencing of RNA-seq

Total RNA from abdominal fat was extracted using the TRIZOL kit protocol (AG21101, Agbio, China), and the quality and concentration of RNA were assessed using NanoDrop 2000. The qualified RNA was used to construct sequencing libraries with Illumina TruSeq® Kit (Illumina, San Diego, USA). Raw data underwent quality control to eliminate adapter sequences and low-quality reads. Therewith, high quality reads were then aligned to chicken genome (GRCg7b, https://www.ncbi.nlm.nih.gov/genome/111v) through HISAT2 to obtain positional information on the reference genome. The above sequencing was completed by Shanghai Personal Biotechnology Co., Ltd.

### Data analysis of RNA-seq

The reads aligned to each gene in each sample were quantified and converted to FPKM values to determine gene expression levels. Differential expression genes (DEGs) were analyzed using DEGseq software, with the threshold set at *P*-value < 0.05 and log2 (fold change) ≥ 1. Subsequently, principal component analysis (PCA) plots and gene clustering heatmaps were generated using R software. DEGs were annotated in the Kyoto Encyclopedia of Genes and Genomes (KEGG, http://www.kegg.jp/) database, with enrichment analysis conducted via ClusterProfiler. Gene set enrichment analysis (GSEA) was performed using the Broad Institute's GSEA platform (http://software.broadinstitute.org/gsea/).

### Library construction and sequencing of ATAC-seq

ATAC-seq was conducted by Wuhan Frasergen Bioinformatics Co., Ltd, the specific steps were as follows: about 50 mg frozen AFT was ground in liquid nitrogen, resuspended and incubated on ice for 10 min, followed by centrifugation to obtain purified nuclei. Then the transposition reaction was performed at 37℃ for 30 min and DNA purification using magnetic beads. Afterward, purified DNA was amplified by PCR. The reaction conditions involved predevaluation by 15 cycles at 72 ℃ for 3 min, 98 ℃ for 30 s and 60 ℃ for 30s. Finally, the library fragment size was assessed using an Agilent 2100, and sequencing was performed on the Illumina platform.

### Data analysis of ATAC-seq

Firstly, quality control of the raw sequencing data in FastQ format was performed using SOAPnuke. The main steps included assessing overall sequencing quality, removing adapter sequences, PCR duplicates and low-quality reads. The Burrows-Wheeler Alignment algorithm was applied to align the clean reads to the chicken reference genome (GRCg7b). Subsequently, the deep tools were used to analyze the distribution of insert fragments within a 3 kb window upstream and downstream of the transcription start site. Differential open chromatin regions were detected with Diffbind, and DESeq2 was employed to assess the differential peaks (DPs) under the condition that *P*-value < 0.05 and log2 (fold change) ≥ 1, and DPs were then annotated for their distribution across genomic functional regions using ChIPseeker. Finally, pathway analysis was conducted through the KOBAS website (http://bioinfo.org/kobas/), and DNA motif enrichment analysis for known transcription factor binding sites was conducted using the JASPAR database to identify the most likely transcription factors based on DPs.

### qRT-PCR

Total RNA from AFT was extracted by Trizol reagent kit (AG21101, Agbio, China), and RNA concentration and purity were determined with Nano Drop 2000 (Nanodrop ND-1000, Thermo Fisher Scientific). Subsequently, cDNA was synthesis from 500 ng RNA using Evo M-MLV RT Master Mix kit (AG11706, Agbio, China). Quantitative analysis of genes was performed with 2×SYBR Green qPCR Master Mix (AG11701, Agbio, China), with detailed procedures referenced from our previous studies (Liu et al., 2024). The primer sequence was shown in Table S1. β-actin served as an internal control to normalize the data for obtaining ΔCT, and 2^−ΔΔCT^ was used to calculate relative expression of the target gene.

### Statistical analysis

All data were presented as Mean ± SEM, and comparisons between different groups were analyzed with the unpaired Student's *t*-test and plotted using GraphPad Prism 8 (San Diego, USA). The *P*-value < 0.05 was considered to be statistically significant, which was indicated as *P-value < 0.05, **P-value < 0.01. The raw sequence data have been deposited in the Genome Sequence Archive (Genomics, Proteomics & Bioinformatics 2021) in National Genomics Data Center (Nucleic Acids Res 2022), China National Center for Bioinformation / Beijing Institute of Genomics, Chinese Academy of Sciences (GSA: , CRA023782) that are publicly accessible at https://ngdc.cncb.ac.cn/gsa/s/RnwxPu2h and https://ngdc.cncb.ac.cn/gsa/s/6WVl4xzV. Prediction of transcription factor binding sites using the JASPAR database.

## Results

### DEG identification of AFT between D14 and D42 in broilers

**Fig. 1:**
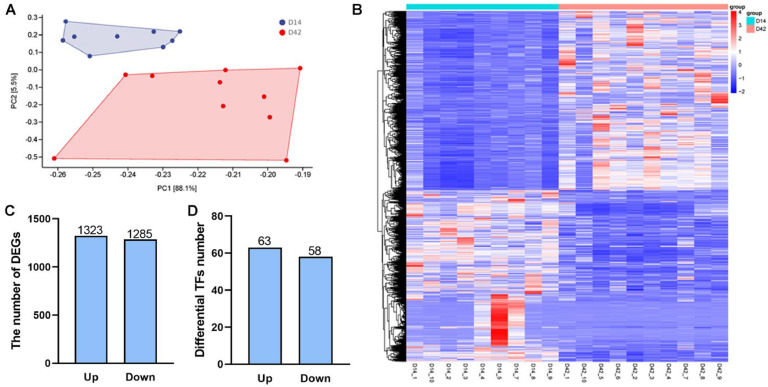
DEG Identification of AFT between D14 and D42 in broilers. (A) Principal components analysis (PCA) of transcriptomes PC1 vs. PC2. (B) Cluster analysis heatmap based on identified DEGs, with up-regulated genes in red color and down-regulated genes in blue color. (C) The number of up-regulated and down-regulated DEGs. (D) The number of up-regulated and down-regulated TFs.

Transcriptional changes of AFT at two physiological stages were elucidated using RNA-seq. Principal component analysis (PCA) showed distinct differences in AFT development at D14 and D42, whereas intra-group similarity was high (Fig. 1A). As shown in Fig. 1B, the heatmap of DEG clustering analysis clearly demonstrated gene expression changes between D14 and D42. The number of up-regulated and down-regulated DEGs at D42 was 1323 and 1285 respectively when compared to D14 (Fig. 1C). Additionally, a total of 121 TFs were identified, including 63 up-regulated and 58 down-regulated at D42 compared to those at D14(Fig. 1D).

### KEGG and GSEA pathway analysis enriched from DEGs

**Fig. 2:**
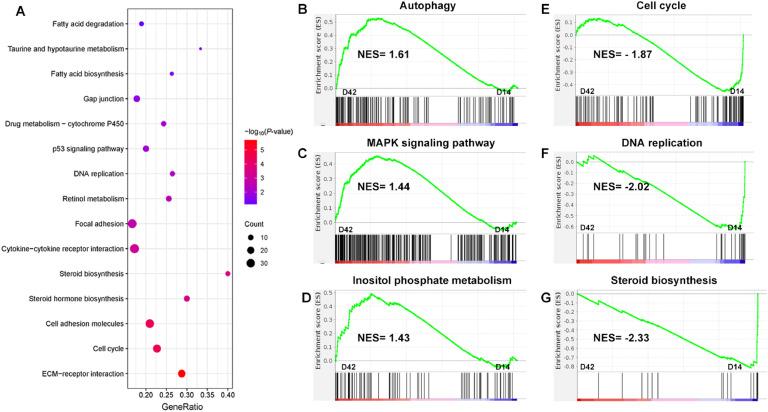
KEGG and GSEA pathway analysis enriched from DEGs. (A) KEGG functional enrichment analysis based on all DEG. (B-G) GSEA analysis of autophagy, cell cycle, MAPK signaling pathway, DNA replication, inositol phosphate metabolism and steroid biosynthesis.

As shown in Fig. 2A, KEGG analysis from total DEGs revealed significant enrichment pathways were connected with adipocytes proliferation, differentiation and lipid metabolism, including DNA replication, cell adhesion molecules, cell cycle, p53 signaling pathway, fatty acid degradation, fatty acid biosynthesis and steroid biosynthesis. Additionally, GSEA analysis indicated upregulation of autophagy, MAPK signaling pathway and inositol phosphate metabolism at D42 compared to D14, whereas cell cycle, DNA replication and steroid biosynthesis were down-regulated (Fig.s 2B-G).

### Chromatin accessibility analysis of AFT between D14 and D42 in broilers

ATAC-seq analysis was used to investigate the differences in chromatin accessibility in AFT at two physiological stages. As illustrated in Fig.s 3A and B, distinct differences were revealed between D14 and D42 based on the heatmaps of correlation analysis among samples and differential peaks. A total of 394 gain and 1195 loss DPs were identified between D14 and D42, which were predicted to be correlated with 319 and 905 genes respectively (Fig.s 3C, D). Lipid metabolic pathways were enriched based on the gain (Fig.s 3E) or loss genes (Fig.s 3F)from DPs, such as FoxO signaling pathway, p53 signaling pathway, PPAR signaling pathway, fat digestion and absorption and glycerolipid metabolism, which were partially consistent with those enriched from DEGs.

**Fig. 3:**
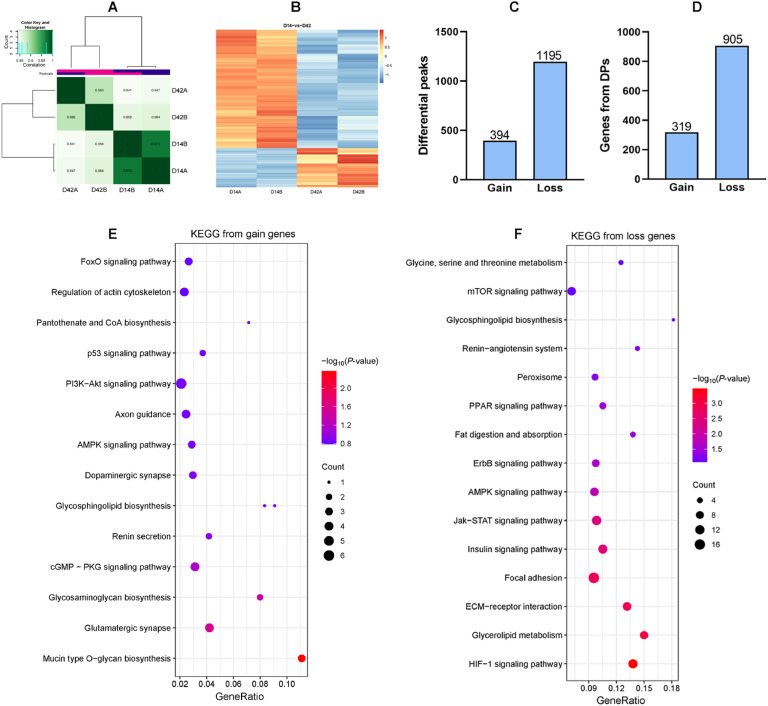
Chromatin accessibility analysis of AFT between D14 and D42 in broilers. (A) Correlation analysis among samples. (B) Heatmap of the differential peaks. (C) The number of gain and loss DPs. (D) The number of gain and loss genes from DPs. (E-F) KEGG pathways analysis enriched based on the gain or loss genes from DPs.

### DNA motifs prediction by integration of RNA-seq and ATAC-seq

To explore the potential regulatory relationship between differential chromatin accessibility and gene transcription in AFT, we performed the intersection analysis between DEGs and predicted genes from DPs. As shown in Fig.s 4A-C, 25 up-regulated DEGs and 75 down-regulated DEGs were overlapped with gain and loss genes form AFT. The comprehensive genes list was provided in Additional file 1. *ACACA, SCD, SREBF1, MIR1682* and *KLF9* genes showed lower expression at D42 (Fig.s 4D-G). In addition, DNA motif analysis for gain and loss peaks was performed, and top 15 gain and loss motifs were displayed in Fig. 5A. Subsequently, we overlapped the loss DNA motifs with the down-regulated TFs from DEGs (Fig. 5B), and identified *NFIX* and *MY*B as potential TFs involved in regulating AFT deposition (Fig. 5C-D). No gain motifs overlapped with up-regulated TFs.

**Fig. 4:**
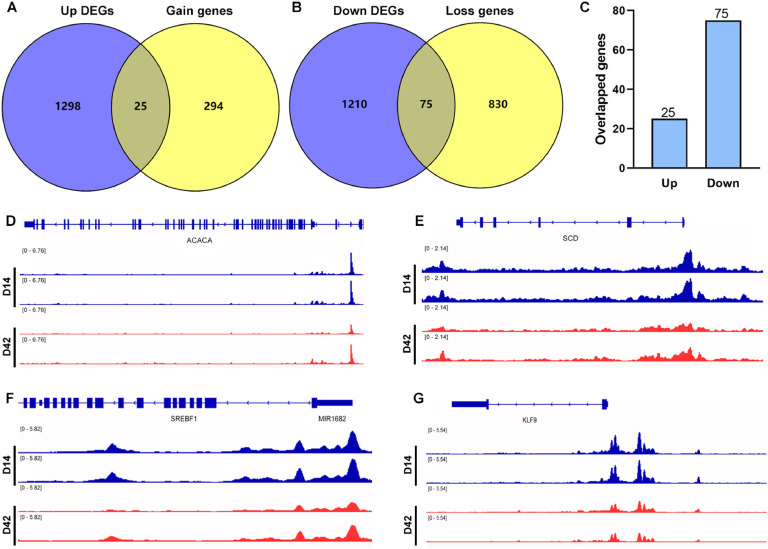
Genes predicted from DEGs overlapped with DPs. (A-B) Venn maps showing the overlap between up- or down-regulated DEGs and predicted genes from corresponding to gain or loss DPs. (C) The number of overlapping genes identified from DEGs and DPs. (D) Signal enrichment of *ACACA, SCD, SREBF1, MIR1682* and *KLF9* in the differential gene regions.

**Fig. 5:**
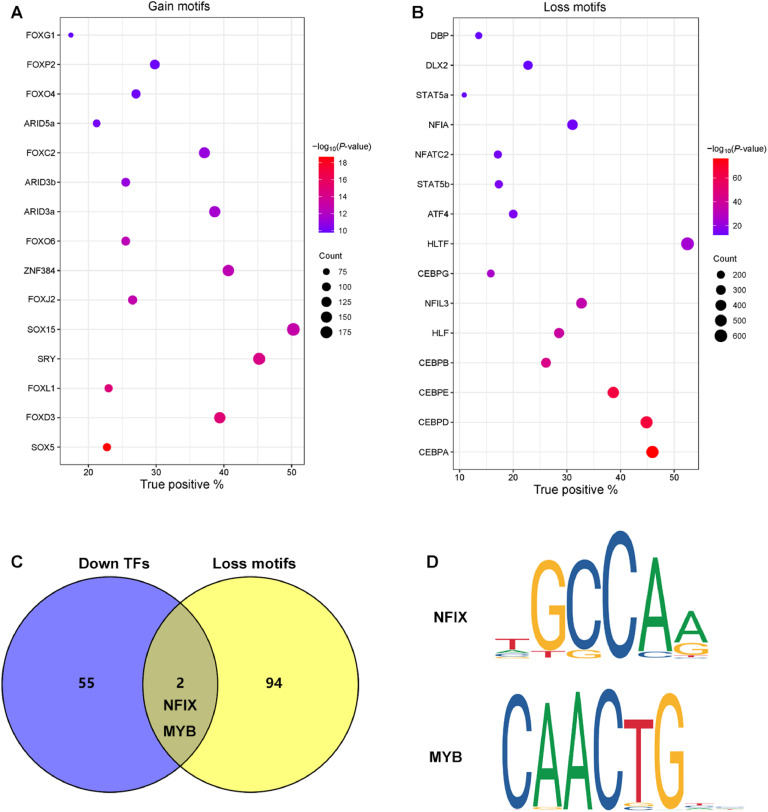
DNA motifs prediction with RNA-seq and ATAC-seq. (A-B) The top 15 gain and loss motifs predicted from differential gene regions. (C) The Venn diagram of down TFs predicted from DEGs overlapped with loss motifs predicted from differential gene regions. (D) DNA sequences of overlapping DNA motifs for *NFIX* and *MYB*.

### Binding sites prediction of MYB and NFIX in promoter regions of lipid metabolism-related genes

**Fig. 6:**
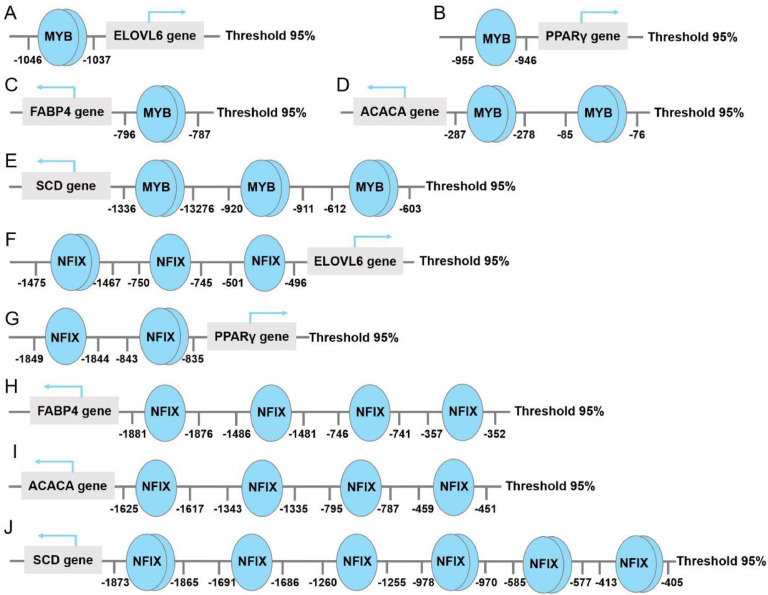
Binding sites prediction of *MYB* and *NFIX* in promoter regions of lipid metabolism-related genes. Binding sites prediction of MYB (A-E) and NFIX (F-J) in the promoter regions of *ELOVL6, PPARγ, FABP4, ACACA* and *SCD*. Blue circles indicate transcription factor binding sites, with positions relative to the transcription start site shown in base pairs. Arrows represent the direction of gene transcription. The threshold 95 % denotes a confidence level above 95 % for binding site prediction.

We predicted the potential binding sites of *MYB* and *NFIX* in the promoter regions of lipid metabolism-related genes. The results indicated that *MYB* binding sites were primarily located in the promoter regions of *ELOVL6* (−1046 bp), *PPARγ* (−955 bp), *FABP4* (−796 bp), *ACACA* (−287 bp) and *SCD* (−1336 bp), suggesting its role as a transcriptional activator in lipid metabolism (Fig. 6A-E). In contrast, *NFIX* binding sites were predominantly positioned upstream of *ELOVL6* (−1475 bp), *PPARγ* (−1849 bp), *FABP4* (−1881 bp), *ACACA* (−1625 bp) and *SCD* (−1873 bp), with multiple binding sites identified in certain promoter regions (Fig. 6F-J), indicating *NFIX* may function as a transcriptional repressor.

### Validation of potential genes regulating adipocyte differentiation

**Fig. 7:**
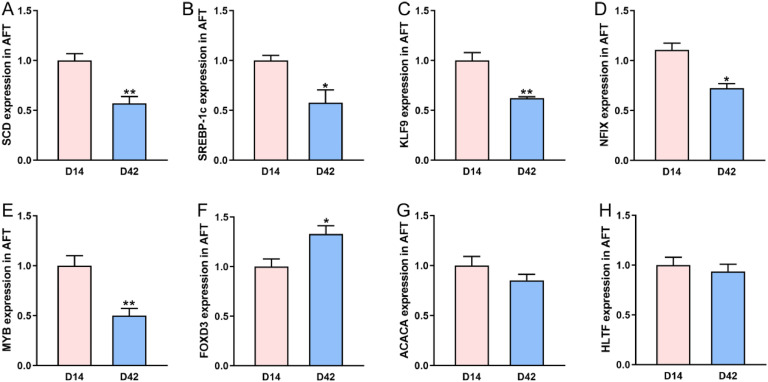
Validation of potential genes regulating adipocyte differentiation. (A-H) Relative mRNA expression of *SCD, SREBP-1c, KLF9, NFIX, MYB, FOXD3, ACACA* and *HLTF*. Data are expressed as mean ± SEM (*n* = 6). Statistical significance is denoted by *P < 0.05 and **P < 0.01.

We quantified the mRNA expression levels of the potential genes involved in regulating adipocyte differentiation using qRT-PCR. Compared to D14, the mRNA expression levels of *SCD, SREBP-1c, KLF9, NFIX* and *MYB* were significantly decreased at D42 (*P* < 0.05, Fig. 7A-E). In contrast, *FOXD3* expression was significantly increased at D42 (*P* < 0.05, Fig. 6F). No significant changes were observed in the expression levels of *ACACA* and *HLTF* (*P* > 0.05, Fig. 7G-H).

## Discussion

Artificial selection for traits such as body weight and growth rate has significantly enhanced production performance but also led to excessive AFT deposition (Wu et al., 2023). It usually requires more energy to deposit fat than protein. Thus, excessive fat deposition reduced feed conversion ratio, resulting in significant economic losses for the poultry industry. Therefore, reducing excessive AFT is critical for the development of poultry industry.

Our previous study investigated the dynamic developmental changes of abdominal fat in broilers and identified D14 might be the crucial physiological stage of adipocyte differentiation (Liu et al., 2023). However, the underlying molecular mechanisms remain unclear. To address this, we analyzed the transcriptional changes of AFT between D14 and D42 through RNA-seq, and identified 2608 DEGs, including 1323 up- and 1285 down-regulated genes. These DEGs were significantly enriched in several pathways related to lipid metabolism, especially fatty acid degradation, fatty acid biosynthesis and steroid biosynthesis. In addition, pathways associated with cell proliferation, such as cell cycle and DNA replication were down-regulated. These results were consistent with previous studies that AFT deposition was driven by both proliferation (hyperplasia) and differentiation (hypertrophy) of adipocytes (Cho et al., 2023), and the adipocyte proliferation activity decreased, whereas the adipocyte hypertrophy was particularly prominent in the accelerating fat deposition during the later growth period of broilers (Liu et al., 2023). Notably, GSEA analysis revealed that autophagy, MAPK signaling and inositol phosphate metabolism were up-regulated at D42. The MAPK signaling pathway played a critical role in various cellular activities, particularly in adipocyte growth and differentiation (Wen et al., 2023). The upregulation of inositol phosphate metabolism suggested enhanced signal transduction activity in fat tissue, likely promoting lipid metabolism and storage (Yu et al., 2023). We speculate that the upregulation of autophagy might be linked to cellular maintenance mechanisms and energy demands, lserving as a response to the demands imposed by lipid metabolism during adipocyte hypertrophy.

To further investigate the transcriptional regulatory mechanisms underlying fat deposition, we performed ATAC-seq analysis to examine changes in chromatin accessibility of AFT at D14 and D42. A total of 394 gain and 1195 loss DPs were identified, and their predicted genes were also enriched in lipid metabolism pathways, including the FoxO signaling pathway, p53 signaling pathway, PPAR signaling pathway, fat digestion and absorption and triglyceride metabolism. These findings suggested that changes in chromatin accessibility may directly influence the activity of these metabolic pathways, thereby regulating fat deposition. The FoxO signaling pathway played a critical role in adipocyte differentiation and energy metabolism (Lee and Dong, 2017), while the p53 signaling pathway was involved in cellular stress responses and metabolic regulation ( Xu et al., 2021; Zhao and Qin, 2022). The PPAR signaling pathway was a well-known regulator of lipid metabolism (Hu et al., 2021), contributing to the synthesis and breakdown of fatty acids. Previous studies have shown that fatty acids serve as essential substrates for lipid synthesis and breakdown, providing energy via β-oxidation and acting as signaling molecules to activate multiple metabolic pathways. Steroids could bind to nuclear receptors to regulate lipogenesis or lipolysis, thereby influencing the storage and utilization of fat. Phosphoinositide played a crucial role in intracellular signal transduction, particularly through the phosphoinositide 3-kinase (PI3K)/Akt signaling pathway, which regulated adipogenesis, insulin sensitivity and lipid transport (Carta et al., 2017; Hannun and Obeid, 2018; Jeon et al., 2023). The interplay between these molecules ensured the maintenance of energy homeostasis and the regulation of key processes in lipid metabolism. Therefore, we hypothesized that changes in chromatin accessibility might regulate these pathways, affecting adipocyte differentiation and lipid metabolism, and ultimately influencing fat deposition in broilers.

Research on chromatin accessibility provides insights into gene regulatory regions associated with AFT deposition (Divoux et al., 2018), offering potential targets for lipid metabolism regulation. RNA-seq quantifies gene expression levels, whereas ATAC-seq assesses chromatin accessibility, which influences transcription factor binding and gene activation. Several studies have combined ATAC-seq with RNA-seq to explore the relationship between chromatin accessibility and transcriptional activity. For instance, an integrated ATAC-seq and RNA-seq analysis in yak adipocyte differentiation revealed that highly expressed genes exhibited significantly enriched ATAC-seq signals near the transcription start site (TSS), supporting the correlation between chromatin accessibility and gene expression (Zhang et al., 2022b). Similarly, a study on pig intramuscular fat (IMF) deposition reported a significant positive correlation (r²= 0.42) between chromatin accessibility and gene expression levels, indicating that chromatin structure changes influence transcriptional activity. However, this correlation is not absolute, as exemplified by THRSP, which exhibited increased chromatin accessibility but decreased mRNA expression, suggesting the involvement of additional regulatory mechanisms (Xu et al., 2022). These findings highlight a general correlation between ATAC-seq and RNA-seq data while emphasizing the complexity of gene regulation. Our study further supports these findings, as ATAC-seq analysis revealed chromatin accessibility changes in lipid metabolism-related genes (such as *ACAACA, SCD, SREBP1* and *KLF9*), which were consistent with their differential expression in RNA-seq. This aligns with previous observation that open chromatin regions frequently overlap with transcriptionally active genes. These genes represent potential regulatory targets for lipid metabolism (Hu et al., 2020; Skrzypski and Kołodziejski, 2023). *ACACA* was a rate-limiting enzyme in fatty acid synthesis, catalyzing the first step in fatty acid synthesis, and its high expression promoted lipid accumulation (Hu et al., 2010). *SCD* was responsible for converting saturated fatty acids into monounsaturated fatty acids, facilitating triglycerides storage in adipocytes ( Feng et al., 2021; Meng et al., 2018). *SREBF1* was a master regulator of lipogenesis, playing a pivotal role in the synthesis of fatty acids and triglycerides (Xu et al., 2018). During adipocyte differentiation, *SREBF1* facilitated lipid accumulation by regulating downstream genes such as *FASN* and *ACACA* (Strable and Ntambi, 2010; Varga et al., 2011). These genes were down-regulated at D42, suggesting that fatty acid synthesis might gradually decrease during the later stages of adipocyte hypertrophy, resulting in a slowdown in lipid accumulation. *KLF9* is a zinc finger transcription factor involved in the regulation of cell proliferation, differentiation and metabolism (Zhu et al., 2024). Whereas its specific role in adipocyte differentiation remains unclear, and *KLF9* might influence adipocyte development by regulating the lipogenesis pathways. In this study, we found that *KLF9* was down-regulated at D42, indicating that it may play a greater role in the early differentiation of adipocyte, with its influence diminishing as the adipocytes mature. There were increasing evidences that genetic variations, miRNAs and epigenetic modifications have significant effects on lipid metabolism (Axsom et al., 2021; Desgagné et al., 2017; Zhang et al., 2022a). As a microRNA, *MIR1682* could inhibit the translation of target genes by binding to mRNA, thus regulating gene expression at the post-transcriptional (Zhang et al., 2016). Although the specific function of *MIR1682* has been studied relatively little in broiler, studies have shown that microRNAs play a key role in adipogenesis and differentiation, potentially regulating lipid synthesis and breakdown by targeting lipid metabolism-related genes.

Changes of gene expression were closely linked to chromatin accessibility (Cao et al., 2023), and it was reported that TFs and other regulatory elements could bind to DNA and initiate gene transcription when the chromatin was in an open state (Schoenfelder and Fraser, 2019). DNA motifs are specific DNA sequences with high affinity for TFs. Therefore, we subsequently performed DNA motif analysis to identify potential TFs based on DPs. We overlapped the loss predicted TFs with the down-regulated TFs from DEGs, and *NFIX* and *MYB* were identified as down TFs. *NFIX* was highly expressed in perirenal fat and brown fat tissues in C57BL/6 J mice, and overexpression of *NFIX* suppressed the mRNA levels of adipogenic genes such as *PPARγ, C/EBPα, aP2* and *adipsin*, thereby inhibiting the differentiation of ST2 cells into adipocytes (Wu et al., 2021). These findings suggested that *NFIX* may play a crucial role as a negative regulator in adipose tissue differentiation. Previous studies have shown that *MYB* plays a key role in the proliferation of adipose-derived stem cells (Yi et al., 2014). In this study, *NFIX* and *MYB* were identified as potential TFs affecting fat development from D14 to D42 in broilers. This suggested their potential roles as negative regulators of adipocyte differentiation and lipid metabolism, which align with previous research indicating that *NFIX* inhibits adipocyte differentiation (Wu et al., 2021). Furthermore, *MYB* as a known regulator of cell proliferation and differentiation (Yi et al., 2024), showed downregulation that may correspond to the stabilization of adipocyte proliferation, with a shift towards hypertrophy mechanisms. DNA motif analysis revealed a set of potential transcription TFs (Avsec et al., 2021; Wang et al., 2021), which might bind to DNA regions associated with genes involved in cell cycle and adipogenesis, thereby influencing AFT deposition. This study leveraged multi-omics techniques to provide preliminarily insights into the molecular mechanisms underlying AFT deposition in broilers. Although multiple potential key genes and regulatory factors have been identified, their specific roles in lipid metabolism still require further validation. Therefore, future research should utilize gene-editing techniques or RNA interference to verify the functions of these genes, enabling a deeper understanding of their involvement in fat deposition.

## Conclusion

In conclusion, this study systematically revealed the dynamic changes in chromatin accessibility and gene expression during AFT development at different growth stages in broilers by integrating RNA-seq and ATAC-seq. We identified several crucial transcription factors, including NFIX and MYB, which may serve as major regulators of AFT deposition. These findings will provide a theoretical basis for investigating the epigenetic modification and transcriptional regulation of AFT development in broilers, and will also be valuable for scientific guidance in breed low-fat broilers.

## Disclosures

The manuscript has been prepared and approved by all authors, with no conflicts of interest. Xiaojun Yang and Yanli Liu provided supervision, resources and participated in the manuscript revision; Xiaoying Liu contributed to drafting the manuscript; Chaohui Wang, Xi Sun and Zhihao Qiao played a key role in conceptualization review and editing.

## Appendix Supplementary materials

Image, application 1
