# Supplementary material for: Identify key transcript factors of adipocyte differentiation in abdominal fat of broilers based on ATAC-seq and RNA-seq
Source: Poult Sci. 2025 Mar 24;104(5):105096. doi: 10.1016/j.psj.2025.105096 (PMC11995140; doi:10.1016/j.psj.2025.105096)
Supplement: Supplementary file 1 [file mmc1.docx]

**Supplemental Table**

Table S1 Forward and reverse primer sequences for qRT-PCR analysis

| Gene | Accession number | Primer sequences, 5' to 3' | Product size, bp |
| --- | --- | --- | --- |
| β-actin | L08165 | F: ATTGTCCACCGCAAATGCTTC | 113 |
|  |  | R: AAATAAAGCCATGCCAATCTCGTC |  |
| SCD | NM_204890 | F: GTTTCCACAACTACCACCATACATT | 175 |
|  |  | R: CCATCTCCAGTCCGCATTTT |  |
| SREBP-1 | XM_046927256 | F: GCCCTCTGTGCCTTTGTCTTC | 130 |
|  |  | R: ACTCAGCCATGATGCTTCTTC |  |
| KLF9 | XM_046936700 | F: CTTCCCCAGTCTGTCGCATT | 177 |
|  |  | R: ACGCTACTGTGGTGTCACTG |  |
| NFIX | NM_001365902 | F: GCCCCTTCCTATGCCTGATT | 179 |
|  |  | R: AGAGGAACCAGGACTGAGACT |  |
| MYB | MF326685 | F: TCCAATGAGACAAGCAGCGA | 169 |
|  |  | R: CAGCTCTCGCATAAACGTCG |  |
| FOXD3 | NM_012183 | F: GCTGCAGCTCAATAGCCTGG | 129 |
|  |  | R: CTCCCATCCCCACGGTACTA |  |
| ACACA | XM_046929960 | F: GCTTCCCATTTGCCGTCCTA | 185 |
|  |  | R: GCCATTCTCACCACCTGATTACTG |  |
| HLTF | NM_001106478 | F: GCCCAAGTGGAAGGGGTAG | 146 |
|  |  | R: TTTGGTGTGGGACCCAACTT |  |
